# Dupilumab‐associated ocular surface disease or atopic keratoconjunctivitis not improved by dupilumab? Upadacitinib may clarify the dilemma: A case report

**DOI:** 10.1002/ski2.354

**Published:** 2024-03-15

**Authors:** Marco Galluzzo, Lorenzo Tofani, Sara Spelta, Marina Talamonti, Alessandra Micera, Luca Bianchi, Marco Coassin, Stefano Bonini, Antonio Di Zazzo

**Affiliations:** ^1^ Department of Systems Medicine University of Rome “Tor Vergata” Rome Italy; ^2^ Dermatology Unit Fondazione Policlinico “Tor Vergata” Rome Italy; ^3^ Ophthalmology Complex Operative Unit University Campus Bio‐Medico Rome Italy; ^4^ Research and Development Laboratory for Biochemical Molecular and Cellular Applications in Ophthalmological Science IRCCS ‐ Fondazione Bietti Rome Italy

## Abstract

Dupilumab‐associated ocular surface disease is a common clinical sign appearing in patients with atopic dermatitis (AD) just few months after dupilumab treatment start, developing in about 25% of patients. Atopic keratoconjunctivitis (AKC) is a well‐identified clinical entity, defined as a chronic inflammatory disease of eye that affects 25%–40% of patients with AD. Most clinical signs of ocular involvement in AD patients treated with dupilumab overlaps the AKC symptoms and signs. We supposed that Dupilumab‐associated ocular surface disease and AKC represent the same disease but differently called by dermatologists and ophthalmologists. AKC‐like disease may develop during dupilumab therapy as a consequence of alternative cytokines pathway activation (e.g. IL33) secondary to IL‐4/13 pathway block. The novel upadacitinib drug may bypass ILs pathway through Janus Kinases selective inhibition, avoiding positive or negative ILs feedback at the ocular surface level. In this case report, molecular analysis on conjunctival samples showed a lower ocular surface inflammation (lower expression of HLADR) although higher levels of IL4 and IL13 in a patient with AD and AKC during upadacitinib therapy, compared to prior dupilumab treatment. Target therapies in patients suffering from AD may prevent ocular and dermatological comorbidities improving quality of life before quality of skin and vision.

## Graphical abstract

In patients with atopic dermatitis under dupilumab treatment, dupilumab‐associated ocular surface disease (DAOSD) is a frequent adverse event. DAOSD is a clear clinical entity defined by ophthalmologists as atopic keratoconjunctivitis (AKC). By blocking pre‐transcriptional pathway upadacitinib may avoid any positive or negative feedbacks or alternative pathways at the ocular surface. Switching systemic therapy to upadacitinib may rapidly reduce ocular inflammation in patients suffering AKC, with quickly improvement of dermatologic manifestations.

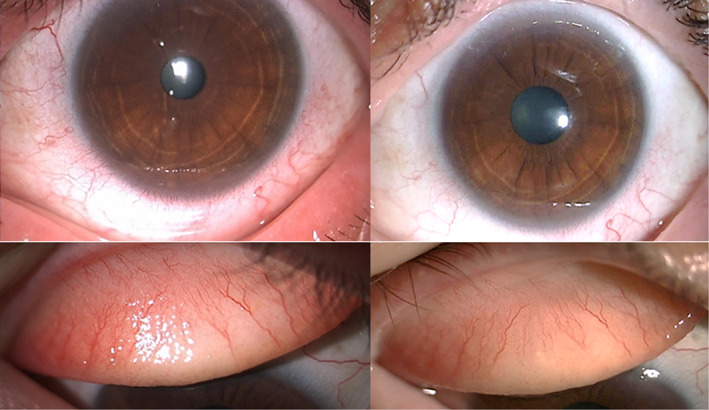

## 1 INTRODUCTION

Atopic dermatitis is a chronic inflammatory pruritic skin disease affecting approximately 3%–5% of adults.^1^

The most common ocular involvement in patients with AD is known as atopic keratoconjunctivitis (AKC).^2^ Ocular manifestations such as recurrent conjunctivitis, keratoconus, anterior subcapsular cataract, Dennie‐Morgan infraorbital fold, and orbital darkening, are considered minor criteria for diagnosis of AD according to the Hanifin‐Rajka Diagnostic Criteria for AD.

Dupilumab, an anti‐interleukin IL‐4Ra antibody inhibiting IL‐4 and IL‐13 broadly used in atopic patients, seems to exacerbate ocular symptoms and signs, including conjunctival hyperaemia, papillary reaction, and superficial punctate keratitis.^3^, ^4^ A 2020 meta‐analysis on 3303 patients demonstrated the efficacy and safety of dupilumab in controlling AD; however, conjunctivitis developed in 26.1% of patients.^1^

Such ocular surface secondary inflammation has been define as DAOSD and its pathogenesis is still poorly understood. Independent risk factors such as baseline AD severity, prior history of conjunctivitis, and local biomarkers (TARC, IgE, and eosinophils) increased risk of conjunctival involvement.^3^, ^5^

Several pathogenic hypothesis for ocular surface disease development have been proposed.

(1) By blocking interleukin 13, dupilumab may cause goblet cells hypoplasia, resulting in decreased mucin secretion, mucosal epithelial barrier dysfunction, and qualitative tear production failure.^6^
(2) Alternatively, upregulation of Th1 response may results as effect of dupilumab on Th2 signalling.^7^
(3) A lower dupilumab bioavailability at the conjunctiva, due to the decreased diffusion of the drug and increased elimination, results in a shorter duration of the effect of dupilumab in the respective part of the eye.
(4) Finally, unmasking of pre‐existent subclinical atopic or allergic inflammatory processes, local immunodeficiency and resulting local infections; increased expression of costimulating proinflammatory molecules (i.e., OX40 L) based on alterations in the immunological milieu; eosinophilia; reduced IL13 related mucus production; disruption of an immune‐mediated response of conjunctival associated lymphoid tissue may be implicated.^4^

Atopic keratoconjunctivitis clinical signs include papillary reaction, conjunctival hyperaemia, mucous filaments, meibomian glands dysfunction, mucocutaneous junction involvement up to trichiasis, punctate superficial keratitis, subepithelial conjunctival fibrosis, symblefaron, corneal neovascularisation and keratinisation.

AKC‐like disease appeared in the first weeks to months of dupilumab treatment and were mild to moderate.^7^

Based on these observations, it is possible that patients with AD who have preexisting ocular disorders may have a lower threshold for the development of severe ocular involvement as an exacerbation of pre‐existing milder conditions in some patients.^3^

Upadacitinib is a novel selective inhibitor of Janus kinase 1 approved for AD patients.

A recent randomized, blinded, multicenter comparator clinical trial of 692 patients with moderate‐to‐severe AD demonstrated the superiority of upadacitinib in a more rapid skin clearance and itch relief with tolerable safety compared with dupilumab, with less outbreak of conjunctivitis (1.4% in upadacitinib group vs. 8.4% in dupilumab group).^8^ Recent evidences showed the improvement of dupilumab‐associated conjunctivitis after switching to upadacitinib.^9^, ^10^

We aim to define clinical and biological ocular inflammation in dupilumab‐induced AKC and the restoration to the prior quiescent ocular surface after switching to upadacitinib therapy.

## 2 CASE REPORT

An ophthalmological assessment was performed for a 54 yo female with severe AD from birth, previously treated with topic and systemic steroids, oral cyclosporine, antihistaminics, and dupilumab (from 2018 to 2023) with little benefits on AD except for head‐neck region and eye involvement. The assessment was performed by a team of specialist ophthalmologists.

Ocular symptoms at baseline (W0) included tearing, itching, foreign body sensation, light sensitivity, swelling, and burning, while ocular signs showed moderate conjunctival and limbal hyperaemia, mucous filaments, superficial punctate keratopathy, tarsal papillary reaction, increased tear meniscus. Conjunctival impression was performed at baseline and after 12 weeks from starting upadacitinib (W12).

Experimental procedures were performed according to guidelines established by the ARVO and adhered to the tenets of the Declaration of Helsinki and were approved by the Intramural Ethical Committee. The patient provided written informed consent to proceed with clinical and biomolecular analysis.

**FIGURE 1:**
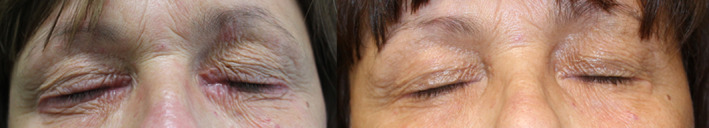
Lids involvement in atopic dermatitis (AD). Cutaneous disease of head‐neck AD patient during dupilumab treatment (on the left) and rapid and complete clinical resolution after 8 weeks upadacitinib treatment (on the right).

**FIGURE 2:**
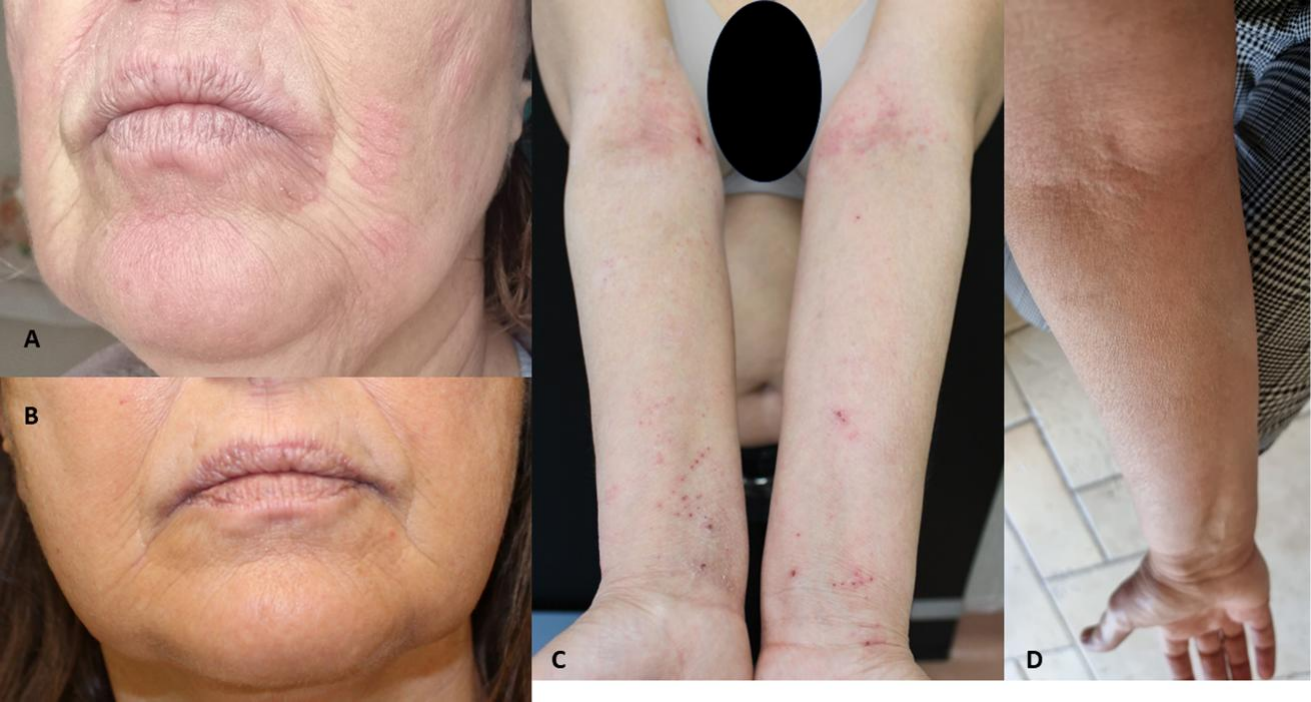
Head‐neck involvement and residual disease on upper limbs during dupilumab treatment (a, c). Disappearance of clinical signs after 8 weeks of upadacitinib treatment (b, d).

**FIGURE 3:**
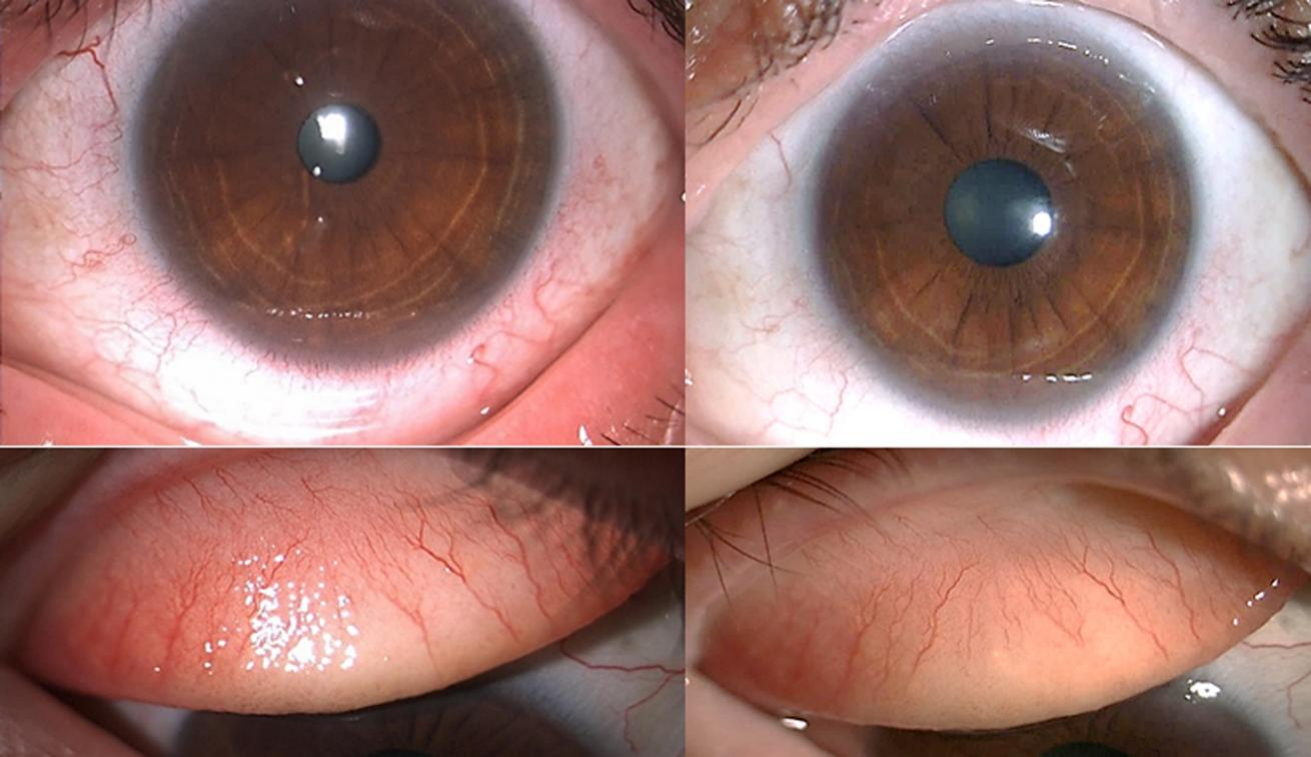
Ocular involvement in atopic dermatitis (AD). The figure shows ocular signs of atopic keratoconjunctivitis (AKC) in a patient previously treated with dupilumab (on the left): conjunctival hyperaemia, mucous filaments, increased tear meniscus, tarsal papillary reaction. On the right, the improvement of hyperaemia and papillary reaction after 12 weeks of treatment with upadacitinib.

At W0 patient was under dupilumab treatment. She stopped dupilumab and after a 12 weeks period of washout, she started upadacitinib 15 mg. After 8 weeks mean benefits involved Body Surface Area (from 20% to 0%), Eczema Area and Severity Index (from 16 to 0), Validated Investigators Global Assessment‐atopic dermatitis (from 3 to 0), Dermatology Life Quality Index (from 12 to 0) and itch‐NRS (from 8 to 0) (Figures 1 and 2). At W12 ocular surface improved with no signs of superficial punctate keratopathy and mucous filaments, no conjunctival hyperaemia, and reduction of papillary reaction (Figure 3).

**FIGURE 4:**
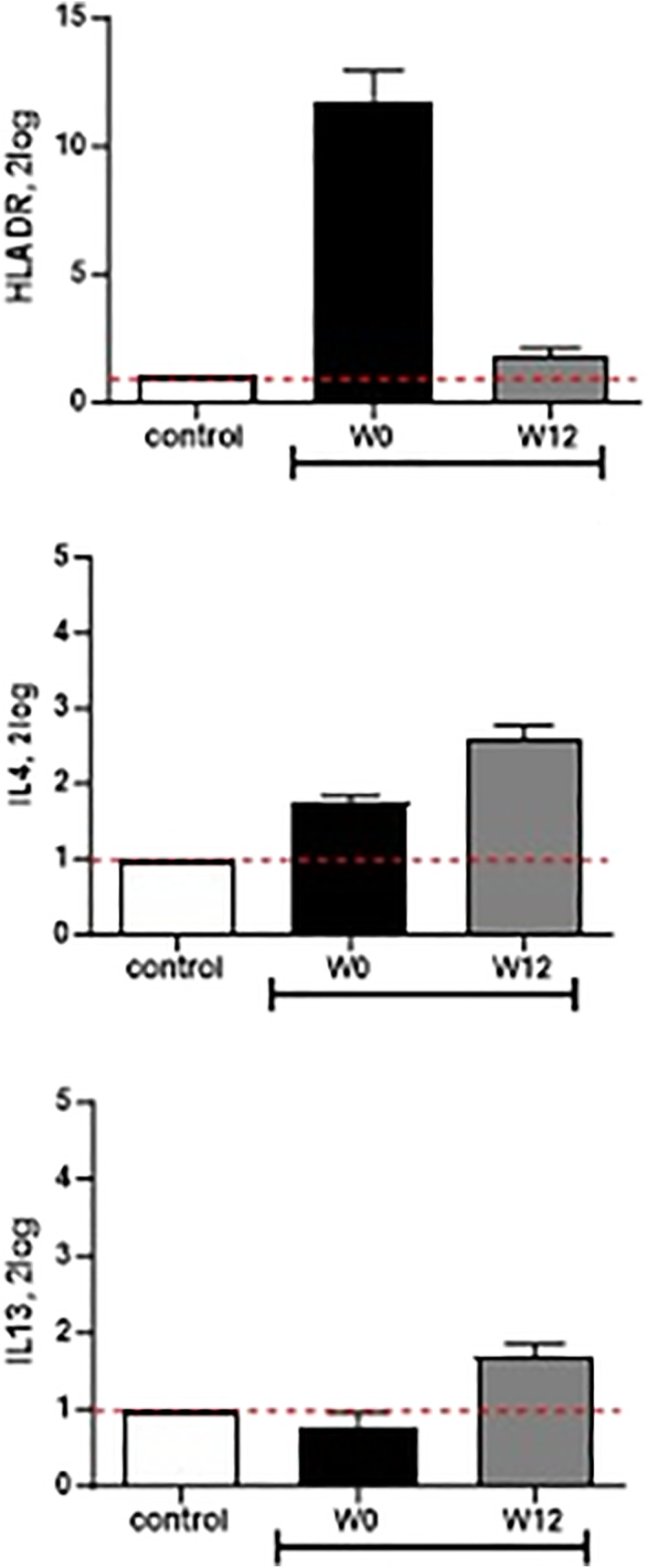
Results of conjunctival impression citology. Molecular analysis on conjunctival cells shows the increase of inflammatory marker HLADR expression during dupilumab treatment (W0) compared to healthy controls and the reduction of the same marker under upadacitinib treatment (W12), despite the increase in IL4 and IL13 values in the same period.

Molecular analysis of conjunctival samples by quantitative RT‐PCR (Table S1) shows a reduction of HLA‐DR expression and an increase of IL4 and IL13 expression from W0 to W12 (Figure 4). Four sex‐ and age‐matched healthy controls have been used as frame of reference.

## 3 DISCUSSION

Most clinical signs of ocular involvement in AD patients treated with dupilumab overlaps the AKC.

In our case report ocular surface disease develops shortly after dupilumab treatment starts. Despite several topic therapies, ocular symptoms and signs do not improve with time. We decided to shift systemic therapy from dupilumab to upadacitinib because of patient's complaint and intolerance to ocular upset. Both dermatological and ophthalmological signs improve just after few weeks after starting the new biological drug, with rapid recovery of patient's symptoms.

Increase in IL4 and IL13 and reduction in HLA‐DR expression showed by molecular results on conjunctival samples entails the post‐transductional action of dupilumab versus pre‐inflammatory cascade inhibition of upadacitinib. An alternative inflammatory pathway may be involved in AKC outbreak if Th2 signalling is blocked by dupilumab. Upadacitinib reduces ocular inflammation, as demonstrated by HLADR expression, despite reactive IL4 and IL13 increase, by selective pre‐transcriptional inhibitory action on Janus Kinases.

AKC‐like disease may develop during dupilumab therapy as a consequence of alternative cytokines pathway activation (e.g. IL33) secondary to IL‐4/13 pathway block. IL‐33 plays important roles in atopic conditions, as recently supposed by Chiricozzi et al.^11^

Atopic keratoconjunctivitis is a severe allergic condition characterised by inflammation affecting the entire ocular surface. Atopic keratoconjunctivitis may cause blindness through corneal neovascularisation and opacities as well as destruction of corneal epithelial stem cells, and cicatricial sequelae.^12^ However, despite strict therapy, such patients experience a critical reduction in their quality of life. Ocular discomfort, itching, and visual impairment limit their daily activities (driving, working, meeting friends) as well as their personal, social, and psychological development in such young patients group.

Atopic keratoconjunctivitis is more common in AD patients with head‐neck involvement.^13^ Discovering risk factors of AKC development during dupilumab treatment can help clinicians in daily practice.^14^ Target therapies in patients suffering from AD may prevent ocular and dermatological comorbidities improving quality of life before quality of skin and vision.

By blocking pre‐transcriptional pathway upadacitinib may avoid any positive or negative feedbacks at ocular surface as well as alternative pathways which may be building up in selective dupilumab IL‐13/IL‐4 antagonism.

However, a definite conclusion needs a wider framework. Main study limitation of all case report involves the sample size.

Future aim tends to understand the molecular basis for the pathogenesis of secondary AKC in a narrow group of patients.

## AUTHOR CONTRIBUTIONS

**Marco Galluzzo**: Conceptualisation (equal); Data curation (equal); Formal analysis (equal); Methodology (equal); Project administration (equal); Supervision (equal); Validation (equal); Visualisation (equal); Writing – review & editing (equal). **Lorenzo Tofani**: Conceptualisation (equal); Data curation (equal); Investigation (equal); Methodology (equal); Resources (equal); Visualisation (equal); Writing – original draft (equal). **Sara Spelta**: Conceptualisation (equal); Data curation (equal); Formal analysis (equal); Investigation (equal); Methodology (equal); Resources (equal); Validation (equal); Visualisation (equal); Writing – original draft (equal). **Marina Talamonti**: Conceptualisation (equal); Data curation (equal); Project administration (equal); Resources (equal); Visualisation (equal). **Alessandra Micera**: Formal analysis (equal); Methodology (equal); Project administration (equal); Resources (equal); Software (equal); Writing – review & editing (supporting). **Luca Bianchi**: Conceptualisation (equal); Project administration (equal); Writing – review & editing (equal). **Marco Coassin**: Conceptualisation (equal); Methodology (equal); Project administration (equal); Writing – review & editing (supporting). **Stefano Bonini**: Conceptualisation (equal); Methodology (equal); Project administration (equal); Writing – review & editing (supporting). **Antonio Di Zazzo**: Conceptualisation (equal); Investigation (equal); Project administration (equal); Supervision (equal); Visualisation (equal); Writing – review & editing (equal).

## CONFLICT OF INTEREST STATEMENT

None to declare.

## ETHICS STATEMENT

Not applicable.

## Supporting information

Table S1

## Data Availability

The data underlying this article will be shared on reasonable request to the corresponding author.
