# Supplementary material for: Dupilumab‐associated ocular surface disease or atopic keratoconjunctivitis not improved by dupilumab? Upadacitinib may clarify the dilemma: A case report
Source: Skin Health Dis. 2024 Mar 15;4(3):e354. doi: 10.1002/ski2.354 (PMC11150752; doi:10.1002/ski2.354)
Supplement: Supplementary file 1 — Table S1 [file SKI2-4-e354-s001.docx]

| Gene | Accession number | Sequences (forward/reverse) | Number of Base pairs |
| --- | --- | --- | --- |
| Inflammatory targets | | | |
| HLADR | NM019111.5 | F: CCT GTC ACC ACA GGA GTG TC  R: GAG AAG AGG CTC ATC CAA GC | 147 |
| IL4 | BC070123.1 | F: TGC ACC GAG TTG ACC GTA AC  R: GTT CCT GTC GAG CCG TTT CA | 198 |
| IL13 | L06801.1 | F: AAC ACG GTC ATT GCT CTC AC  R: GCT GTC AGG TTG ATG CTC CA | 164 |
| Reference genes | | | |
| H3 | NM005324.4 | F: GTC TGC AGG CTG GCA TAG AAG  R: TCG CCT TCT GGG TTG AGT G | 110 |
| β2MG | NM004048 | F: CCT GGA GGC TAT CCA GCG TA  R: CGG ATG GAT GAA ACC CAG AC | 110 |
| GAPDH | BC013310.2 | F: CCT GAC CTG CCG TCT AGA AA  R: ACC TGG TGC TCA GTG TAG CC | 111 |

## Table S1. Molecular analysis: primers and amplifications

1. Primers (accession number) were designed one intron spanning (<https://bioinfo.ut.ee/primer3-0.4.0/>) from human mRNA complete sequence available at: <https://www.ncbi.nlm.nih.gov/gene> with 50-60% GC-content and >61°C annealing temperature.
2. cDNA Synthesis was carried out with 5X RT Buffer (DTT), RNasin, dNTPs and Random Primer Mix at 40°C for 45min (cDNA Synthesis Kit plus gDNA Eraser; FS-RT-1023).
3. The specific amplification was performed in a 48-Eco-Illumina real time PCR platform (Illumina Way, San Diego, CA): a single step for enzyme activation (2min^/^95°C) followed by 39 amplification cycles consisting of a denaturation (15sec^/^94°C) and specific annealing (61-63°C/30sec) steps (SUPER GREEN qPCR 2X Master Mix protocol). Melting curve was registered from 65.0°C to 94.1°C, according to the standard protocol. Specific amplifications were tested by verifying the single curve specific for each amplicon (bps).

All reagents were from FMB (Fisher Molecular Biology, Trevose, PA, USA) and Promega Corporation (Madison, Wisconsin, USA).
